# eIF5A Promotes Translation Elongation, Polysome Disassembly and Stress Granule Assembly

**DOI:** 10.1371/journal.pone.0009942

**Published:** 2010-04-01

**Authors:** Chi Ho Li, Takbum Ohn, Pavel Ivanov, Sarah Tisdale, Paul Anderson

**Affiliations:** Division of Rheumatology, Immunology and Allergy, Brigham and Women's Hospital, Boston, Massachusetts, United States of America; University of Washington, United States of America

## Abstract

Stress granules (SGs) are cytoplasmic foci at which untranslated mRNAs accumulate in cells exposed to environmental stress. We have identified ornithine decarboxylase (ODC), an enzyme required for polyamine synthesis, and eIF5A, a polyamine (hypusine)-modified translation factor, as proteins required for arsenite-induced SG assembly. Knockdown of deoxyhypusine synthase (DHS) or treatment with a deoxyhypusine synthase inhibitor (GC7) prevents hypusine modification of eIF5A as well as arsenite-induced polysome disassembly and stress granule assembly. Time-course analysis reveals that this is due to a slowing of stress-induced ribosome run-off in cells lacking hypusine-eIF5A. Whereas eIF5A only marginally affects protein synthesis under normal conditions, it is required for the rapid onset of stress-induced translational repression. Our results reveal that hypusine-eIF5A-facilitated translation elongation promotes arsenite-induced polysome disassembly and stress granule assembly in cells subjected to adverse environmental conditions.

## Introduction

Eukaryotic initiation factor 5A (eIF5A) has been implicated in multiple cellular functions including translation initiation [1]–[3], mRNA decay [4], cell cycle progression [5], [6], cell survival [7], [8], retroviral infection [9] and translation elongation [10], [11]. eIF5A was first described as a ribosome-associated translation initiation factor purified from rabbit reticulocytes [1]. In reticulocyte lysates, eIF5A is required for methionyl-puromycin, but not globin, synthesis from a globin reporter transcript [1]. In *Saccharomyces cerevisiae*, depletion of eIF5A reduces global protein synthesis by about 30%, suggesting that it has an accessory, non-essential, role in protein synthesis [12]. Under these conditions, accumulation of free 60S ribosomal subunits suggests a possible role in a late stage of translation initiation [12]. Moreover, mutational analysis using human eIF5A to complement an eIF5A null strain of S. cerevisiae supports a role for eIF5A in protein synthesis [3], [13]. These studies show that mutants deficient in protein synthesis are also deficient in cell growth. Taken together, these studies suggest that eIF5A may function as a non-essential translation initiation factor.

Two recent studies have implicated hypusine-eIF5A in the process of translation elongation in *S. cerevisiae* [10], [11]. Temperature sensitive eIF5A mutants reduce the rate of ^35^S-methionine incorporation, a consequence of prolonged ribosome transit time. In vitro translation assays using yeast extracts confirmed that recombinant eIF5A, but not a non-hypusinatable mutant (eIF5A(K51R)), enhances the rates of elongation and termination [11]. The ability of the eEF2 inhibitor sordarin to promote the growth defect of eIF5A mutants suggested that eIF5A and eEF2 might cooperatively promote translation elongation [11].

eIF5A is the only known protein to be modified by hypusine, a spermidine derivative that is added via a two-step enzymatic process [14]. In the first step, a 4-amino-butyl group from spermidine is added to lysine residue 50 by deoxyhypusine synthase. In the second step, the 4-amino-butyl group is hydroxylated by deoxyhypusine hydroxylase. Hypusine modification is essential for cell survival as an eIF5A (K50R) mutant that is not modified by hypusine fails to rescue eIF5A deletion mutants in Saccharomyces cerevisiae [15]. Hypusine modification is also required to promote interactions with the translation machinery, enhance protein synthesis and stimulate cellular growth [14].

Translation initiation factors play important roles in the regulation of stress-induced translational arrest. Stress-induced phosphorylation of eIF2α reduces the availability of the eIF2α/tRNA_i_^Met^/GTP ternary complex responsible for initiation codon recognition. Under these conditions, assembly of a translationally stalled, non-canonical 48S preinitiation complex disassembles polysomes and promotes the aggregation of untranslated mRNPs at discrete foci known as stress granules (SGs) [16]. The core constituents of SGs include components of the 48S preinitiation complex as well as an eclectic group of proteins whose sequestration may regulate the survival of stressed cells [16]. In addition, the assembly of SGs helps to reprogram protein expression in a way that promotes cell survival under adverse conditions [17]. We recently described an siRNA screen that identified proteins involved in the assembly of SGs [18]. This screen implicated ornithine decarboxylase, a component of the polyamine synthetic pathway, in the assembly of SGs. Here we report that hypusine-modified eIF5A has modest effects on normal protein synthesis, but is required for the rapid polysome disassembly and translational repression observed in cells subjected to arsenite-mediated oxidative stress. We show that eIF5A promotes ribosome run-off indicating that it supports translation elongation in cells exposed to adverse environmental conditions.

## Results

### The polyamine pathway is required for the assembly of SGs and PBs

**Figure 1:**
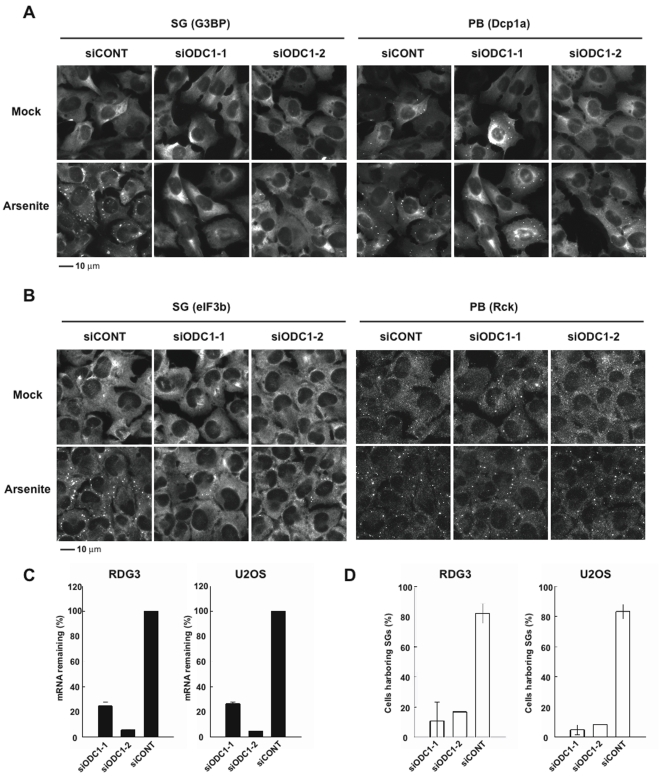
Depletion of ornithine decarboxylase (ODC) inhibits SG assembly. (A) RDG3 (GFP-G3BP;RFP-Dcp1a) double stable cells were transfected with control (siCONT) or ODC-specific (siODC1) siRNAs, then cultured in the absence (Mock) or presence (Arsenite) of 120 µM sodium arsente for 30 min before processing for immunofluorescence microscopy. G3BP-GFP was used to visualize SGs (left panels), DCP1a-RFP was used to visualize PBs (right panels). Scale bar, 10 µm. (B) U2OS cells were transfected with control (siCONT) or ODC-specific (siODC1) siRNAs, then cultured in the presence of 120 µM arsenite for 30 min before processing for immunofluorescence microscopy using antibodies reactive with eIF3b (SG marker) and Rck (PB marker). Scale bar, 10 µm. (C) Knockdown efficiency of *ODC1* mRNA was assessed using RT-qPCR in both RDG3 and U2OS cells. Results are reported as means ± S.D., n = 4. (D) Graphical representation of SG assembly in RDG3 and U2OS cells transfected with control (siCONT) or non-overlapping ODC1-specific (siODC1-1 and siODC1-2) siRNAs prior to treatment with 120 µM arsenite for 30 min. Cell counting data are shown as means ± S.D. from different experiments (siODC1-1, n = 2; siODC1-2, n = 1 for each cell line).

We recently completed an siRNA screen designed to identify genes involved in the assembly of SGs and processing bodies (PBs) [18]. Knock down of ornithine decarboxylase (ODC), an enzyme required for polyamine (e.g., spermine and spermidine) synthesis, was found to inhibit the assembly of SGs and PBs in a primary screen using pooled oligonucleotides. Because only one out of four individual siRNAs produced this phenotype, ODC did not make our list of SG/PB regulatory genes [18]. In follow up experiments, two additional non-overlapping siRNAs targeting ODC were found to inhibit SG and PB assembly prompting a further evaluation of this target. Figure 1A shows that ODC knockdown strongly inhibits the assembly of arsenite-induced SGs (GFP-G3BP) in RDG3 cells (U2OS cells stably transfected with GFP-G3BP and RFP-DCP1a). Similar results were obtained using U2OS cells that were stained with anti-eIF3b (SG specific) and anti-Rck (PB selective) (Figure 1B). In both cases, more modest inhibition of PBs (RFP-DCP1a or anti-Hedls) was observed (Figure 1A and 1B, right panels). The knockdown efficiency of ODC1 mRNA produced by non-overlapping specific (ODC1-1 and ODC1-2) or control siRNAs was quantified using quantitative real time PCR (RT-qPCR) and depicted in Figure 1C. The average inhibition of arsenite-induced SGs produced by ODC1 specific and control siRNAs is shown in Figure 1D. These results implicate the polyamine pathway in the arsenite-induced assembly of SGs and PBs.

### Hypusine-modified eIF5A contributes to SG assembly

**Figure 2:**
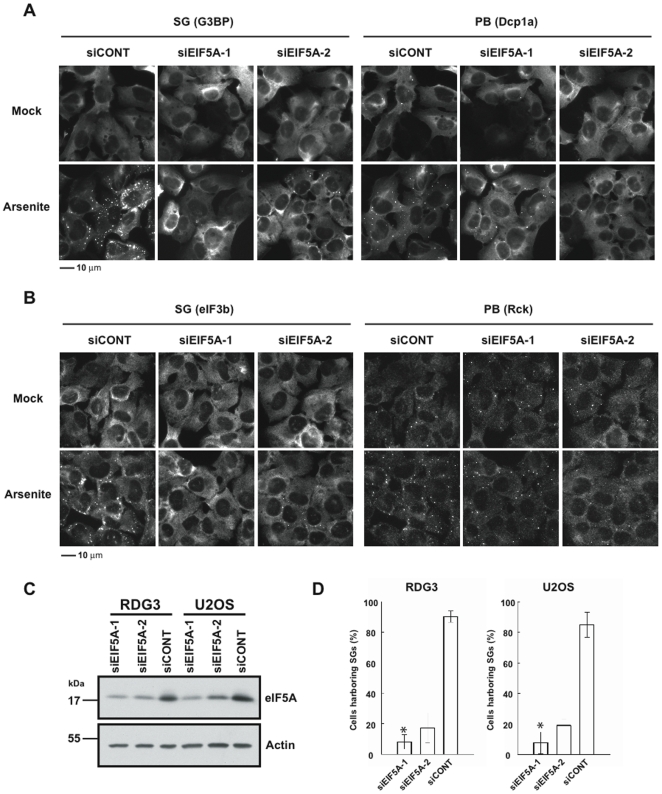
Depletion of eIF5A inhibits SG assembly. (A) RDG3 cells were transfected with control (siCONT) or eIF5A-specific (siEIF5A-1 and siEIF5A-2) siRNAs, then cultured in the absence (Mock) or presence (Arsenite) of 120 µM arsenite for 30 min before processing for immunofluorescence microscopy. G3BP-GFP was used to visualize SGs (left panels), DCP1a-RFP was used to visualize PBs (right panels). Scale bar, 10 µm. (B) U2OS cells were transfected with control (siCONT) or eIF5A-specific (siEIF5A) siRNAs, then cultured in the absence (Mock) or presence (Arsenite) of 120 µM arsenite for 30 min before processing for immunofluorescence microscopy using antibodies reactive with eIF3b (SG marker) and Rck (PB marker). Scale bar, 10 µm. (C) Western blot analysis quantifying the knockdown efficiency of eIF5A in both U2OS and RDG3 cells (upper panel). Actin was used as loading control (lower panel). (D) Graphical representation of SG assembly in RDG3 and U2OS cells transfected with control (siCONT) or non-overlapping EIF5A-specific (siEIF5A-1 and siEIF5A-2) siRNAs prior to treatment with 120 µM arsenite for 30 min. Cell counting data are shown as means ± S.D. from different experiments. *, *P*<0.01 (siEIF5A-1, n = 4 (RDG3) and n = 3 (U2OS); siEIF5A-2, n = 2 for each cell line).

The polyamine pathway is required for the hypusine modification of eIF5A [1]–[3]. We found that two non-overlapping siRNAs (siEIF5A-1, siEIF5A-2) targeting eIF5A strongly inhibit arsenite-induced assembly of SGs in RDG3 cells (Figure 2A and 2D). This finding was confirmed using U2OS cells that were stained with antibodies reactive with eIF3b (SG marker) and Rck (PB marker) (Figure 2B and 2D). The knockdown efficiency of eIF5A was quantified by western blot analysis in both RDG3 and U2OS cells (Figure 2C). It has recently been shown that depletion of eIF5A in yeast results in a significant decrease in PB formation (Gregio et al., 2009). In RDG3 cells, eIF5A knockdown has a modest effect on the assembly of constitutive and stress-induced PBs (Figure 2A, right panels). In U2OS cells processed for immunofluorescence microscopy using Rck as a PB marker, eIF5A knockdown has little or no effect on PB assembly (Figure 2B, right panels and Figure S1A). Interestingly, eIF5A knockdown inhibits the assembly of arsenite or thapsagargin-induced, but not pateamine A- or clotrimazole-induced SGs (Figure S2). The molecular distinctions that determine the requirement for eIF5A in the assembly of SGs remain to be determined.

**Figure 3:**
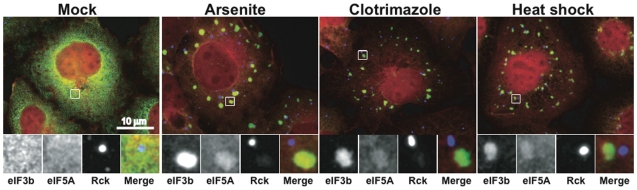
eIF5A is concentrated at SGs. COS7 cells were treated with sodium arsenite (0.5 mM), clotrimazole (20 µM) or heat shock (44°C) for 1 hour prior to processing for immunofluorescence microscopy using antibodies reactive with eIF3b (green, SG marker), Rck (blue, PB marker), and eIF5A (red). Enlarged views of boxed areas show individual channels and merged views.

Several translation initiation factors are components of the stalled translation initiation complex that is concentrated at SGs. We used immunofluorescence microscopy to determine the subcellular localization of eIF5A in COS7 cells cultured in the absence or presence of arsenite, clotrimazole or heat. In unstressed cells, eIF5A has a diffuse cytoplasmic distribution (Figure 3, Mock). In stressed cells, eIF5A is partially relocalized to eIF3b-containing cytoplasmic SGs but excluded from Rck-containing cytoplasmic PBs (Figure 3, Arsenite; Clotrimazole; Heat shock). Thus, eIF5A is a component of the stalled translation initiation complex that accumulates at SGs.

**Figure 4:**
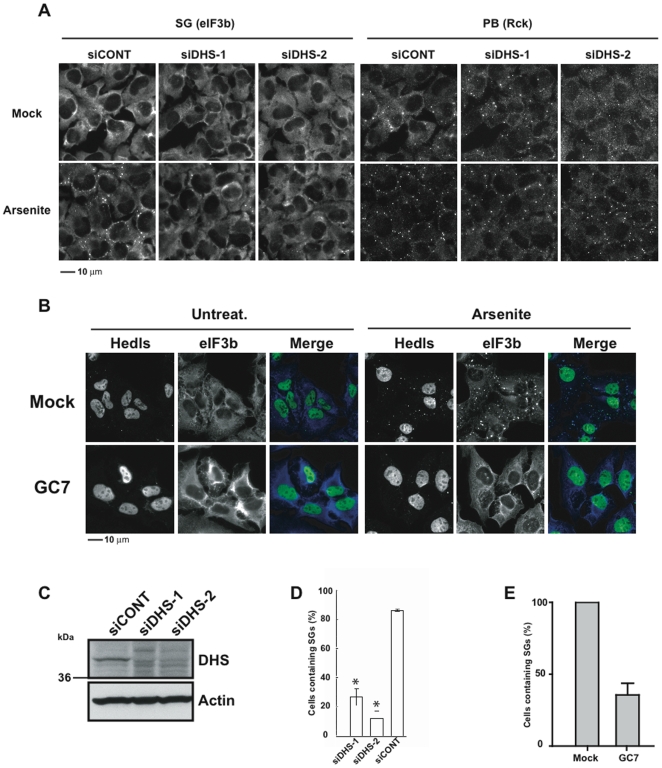
Hypusination of eIF5A is required for SG assembly. (A) U2OS cells transfected with control (siCONT) or DHS-specific (siDHS-1 and siDHS-2) siRNAs were cultured in the absence (Mock) or presence (Arsenite) of 120 µM arsenite for 30 min before processing for immunofluorescence microscopy using antibodies reactive with eIF3b (SG marker) and Rck (PB marker). Scale bar, 10 µm. (B) Hypusination inhibitor GC7 inhibits SG assembly. U2OS cells were treated with GC7 (10 µM) or vehicle control (Mock) for 48 hours prior to culture in the absence (Untreat) or presence (Arsenite) of 0.1 mM sodium arsenite for 1 hour, then processed for immunofluorescence microscopy using antibodies reactive with Hedls (PB marker) and eIF3b (SG marker). Merged views are shown in the right panels. Scale bar, 10 µm. (C) Western blot analysis quantifying the knockdown efficiency of DHS (upper panel). Actin was used as loading control (lower panel). (D) Graphical representation of SG assembly in U2OS cells transfected with control (siCONT) or non-overlapping DHS-specific (siDHS-1 and siDHS-2) siRNAs prior to treatment with 120 µM arsenite for 30 min. Cell counting data are shown as means ± S.D. from different experiments.*, p<0.05; **, p<0.01 (siDHS-1, n = 3; siDHS-2, n = 2). (E) U2OS cells cultured with vehicle (Mock) or GC7 (10 µM) as in (B). The percentage of cells with SGs was quantified microscopically and graphed as an average ± S.D. (n = 2).

**Figure 5:**
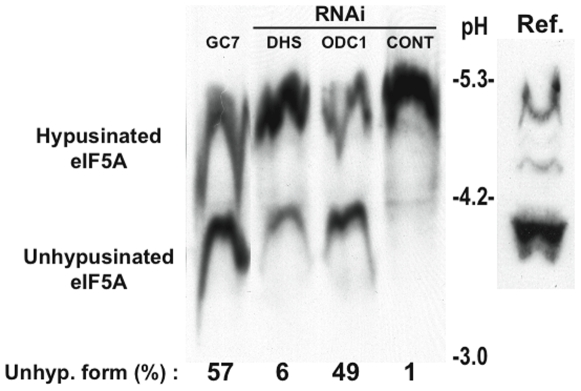
Isoelectric focusing analysis quantifies eIF5A hypusination. U2OS cells were transfected with control (CONT), DHS-specific (DHS), or ODC1-specific (ODC1) siRNAs or treated with GC7 (10 µM) for 1 hour prior to processing for IEF analysis. U2OS cells expressing recombinant eIF5A (K50A) was used as a reference standard for unhypusinated eIF5A. The percentage of unhypusinated eIF5A in each sample was quantified using densitometric analysis.

The activity of eIF5A is modulated by the covalent addition of the spermidine derivative hypusine to lysine residue 50. Knockdown of deoxyhypusine synthase (DHS), an enzyme required for the conversion of spermidine to hypusine, inhibits the assembly of SGs, but not PBs, in U2OS cells (Figure 4A: siDHS-1 and siDHS-2 target two non-overlapping sites in the 3′ untranslated region of the DHS transcript). The knockdown efficiency of DHS was quantified using RT-qPCR (data not shown) and western blot analysis (Figure 4C). Quantification of the effect of DHS knockdown on the arsenite-induced assembly of SGs is depicted in Figure 4D. Thus, the importance of ODC in SG assembly may be to provide a spermidine substrate for hypusine modification of eIF5A. In support of this hypothesis, N1-guanyl-1,7-diaminoheptane (GC7), a chemical inhibitor of DHS [19], eliminates arsenite-induced SGs (Figure 4B, eIF3b), and partially reduces arsenite-induced PBs (Figure 4B, Hedls). Quantification of the effect of GC7 on the arsenite-induced assembly of SGs is depicted in Figure 4E. In cells treated with 20 µM GC7 for 24 hours, the assembly of SGs, but not PBs, is inhibited (Figure S1B). After 48 hours, PB assembly is also affected, but the appearance of cell vacuoles suggests that this may be due to toxic effects of the drug. To confirm that these interventions inhibit hypusine modification of eIF5A, we used isoelectric focusing analysis to quantify the relative expression of hypusine-modified and unmodified eIF5A (Figure 5). Lysates from U2OS (eIF5A(K50A)) transfectants were used as a reference standard to mark the migration of endogenous (hypusine-modified) and recombinant (unmodified) eIF5A (Figure 5, [13]). In cells treated with a control siRNA, eIF5A is quantitatively modified with hypusine (Figure 5, CONT). Knock down of either ODC or DHS, or treatment with GC7, results in accumulation of varying amounts of unmodified eIF5A. Although the extent to which these treatments prevent hypusine modification does not precisely correlate with their ability to inhibit SG assembly (Figures 1D, 4D and 4E), this may reflect experimental variation.

### Hypusine-modified eIF5A differentially modulates translation in the absence or presence of stress

**Figure 6:**
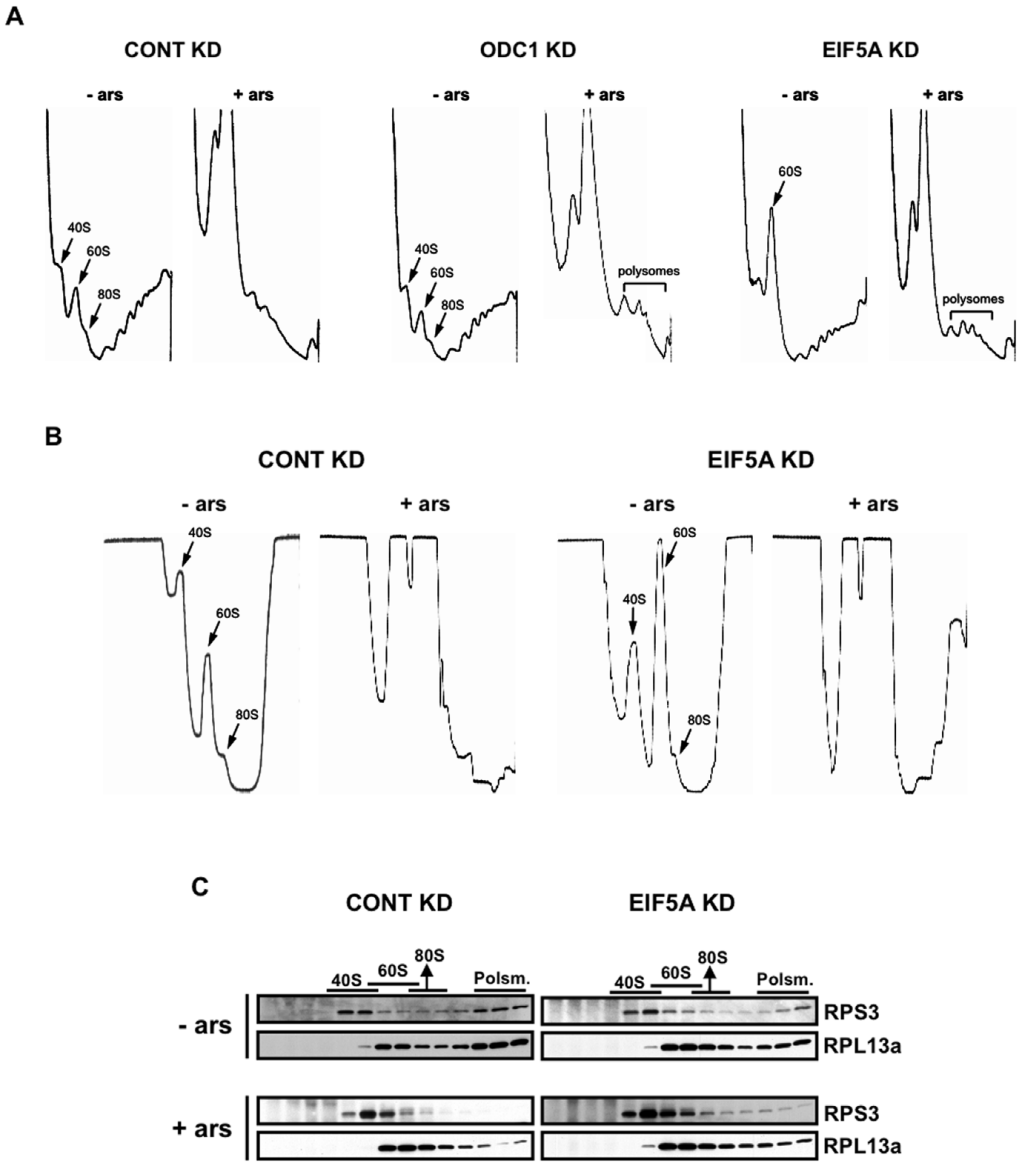
Knockdown of ODC1 or eIF5A inhibits stress-induced polysome disassembly. (A) U2OS cells were transfected with control (CONT KD), ODC1-specific (ODC1KD) or eIF5A-specific (EIF5A KD) siRNAs, then cultured in the absence (−ars) or presence (+ars) of 0.1 mM sodium arsenite before processing for sucrose gradient analysis. The migration of 40S, 60S and 80S ribosomes is indicated by arrows. The migration of preserved low density polysomes is indicated by brackets. (B) The analysis described in (A) was repeated using prolonged centrifugation times and more sensitive readings to improve the resolution of ribosomal subunits. (C) Fractions from control and eIF5A knockdown sucrose gradients were analyzed by Western blotting using antibodies reactive with RPS3 and RPL13a.

We used sucrose gradient analysis to compare polysome profiles in cells expressing reduced levels of ODC or eIF5A. U2OS cells were treated with control, ODC or eIF5A targeted siRNAs, then cultured in the absence or presence of arsenite (0.1 mM) for 1 hour prior to harvesting for sucrose gradient fractionation. In control cells, arsenite-induced translational arrest results in the collapse of polysome profiles and accumulation of monosomes and individual ribosomal subunits (Figure 6A, left panel). Knockdown of ODC has no effect on the polysome profile from cells cultured in the absence of stress, but arsenite-induced polysome collapse is partially inhibited resulting in the retention of low-density polysomes and the accumulation of reduced amounts of monosomes and ribosomal subunits (Figure 6A, middle panel). Knockdown of eIF5A modestly reduces the accumulation of polysomal RNPs and increases the accumulation of ribosomes or ribosomal subunits in the absence of stress (Figure 6A, right panel). Arsenite-induced polysome collapse is inhibited to an extent that is similar to that observed following ODC knockdown. Thus, both ODC and eIF5A are required for efficient polysome disassembly in cells treated with arsenite. When analyzed using sucrose gradient conditions that optimize visualization of ribosomal subunits, it is clear that knockdown of eIF5A results in accumulation of 60S ribosomal subunits in unstressed cells (Figure 6B and 6C). A similar phenotype has been described following knockdown of eIF6, an initiation factor involved in ribosomal subunit joining [20].

**Figure 7:**
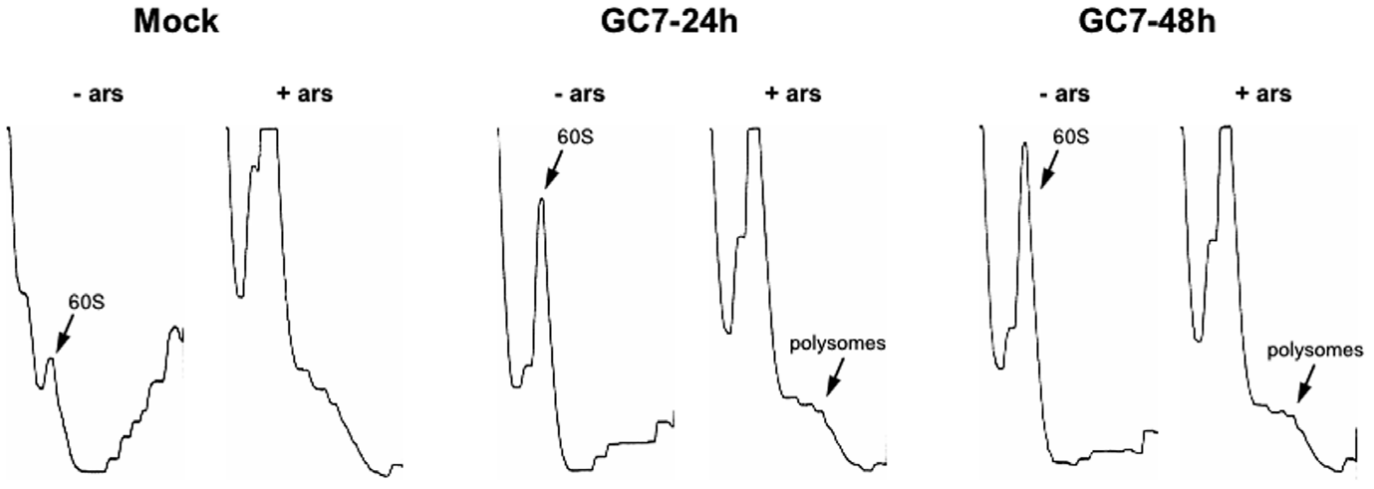
GC7-mediated hypusination inhibition phenocopies eIF5A knockdown. U2OS cells were treated with GC7 (10 µM) or vehicle control (Mock) for the indicated times before culturing cells in the absence (−ars) or presence (+ars) of 0.1 mM sodium arsenite for 1 hour and processing for sucrose gradient analysis. The migration of 60S ribosomal subunits and preserved low density polysomes are indicated.

Sucrose gradient analysis of U2OS cells treated with the DHS inhibitor GC7 prior to culture in the absence or presence of arsenite reveals polysome profiles that phenocopy eIF5A knockdown (Figure 7). Although the specificity of chemical inhibitors is always suspect, this result supports our contention that hypusine-modified eIF5A affects ribosomes in unstressed cells and is required for stress-induced polysome disassembly.

**Figure 8:**
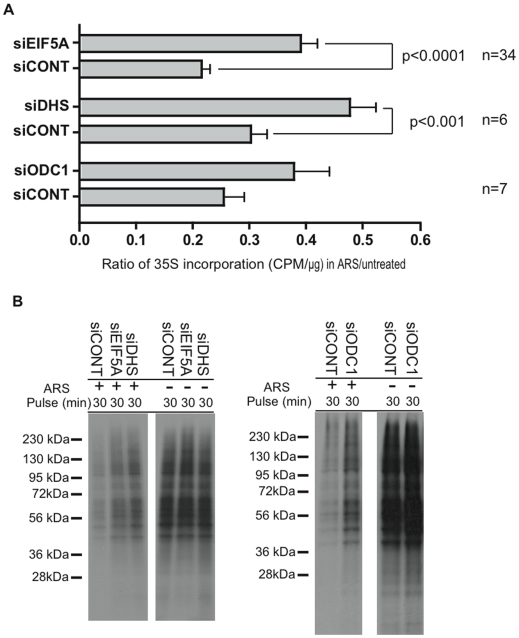
Hypusine-modified eIF5A is required for stress-induced translational arrest. (A) U2OS cells transfected with control (siCONT), DHS-specific (siDHS), or ODC1-specific (siODC1) siRNAs were cultured in the absence or presence of 0.1 mM sodium arsenite for 30 minutes, then pulsed with ^35^S-methionine labeling medium for an additional 30 minutes. Cells were washed and acetone precipitated to quantify ^35^S-methionine incorporation. The ratio of ^35^S-methionine incorporation (CPM/µg protein) in arsenite treated (ARS) vs. untreated cells is reported as mean ± (S.E) from the indicated number of experiments. (B) SDS-PAGE analysis of metabolically labeled protein in cell extracts with or without sodium arsenite treatment. Equivalent amounts of total protein were loaded in each lane.

We used metabolic pulse labeling with ^35^S-methionine to compare the rates of protein synthesis in U2OS cells treated with control or specific siRNAs. Cells were cultured in the absence or presence of arsenite (0.1 mM) for 30 minutes, then pulsed with ^35^S-methionine for 30 minutes before quantifying ^35^S-incorporation. Knockdown of eIF5A modestly inhibits ^35^S-methionine incorporation in the absence of stress (95±3% of control levels, n = 29). Knockdown of eIF5A has a greater effect on protein synthesis in stressed cells, however. The ratio of ^35^S-methionine incorporation in arsenite-treated/untreated cells (i.e., percent arsenite-induced inhibition of protein synthesis) following knockdown with the indicated siRNAs is quantified in Figure 8A. Reduced expression of eIF5A significantly abrogates arsenite-induced translational repression (Figure 8A, upper bars). This was observed using three different siRNAs targeting non-overlapping regions of eIF5A transcripts. Reduced expression of DHS also significantly abrogates arsenite-induced translational repression (Figure 8A, middle bars), suggesting that hypusine-eIF5A promotes stress-induced translational arrest. A similar trend (not statistically significant) was observed in cells treated with siRNAs targeting ODC (Figure 8A, lower bars). eIF5A, DHS, and ODC1 have a global effect on stress-induced translational repression as revealed in autoradiographs prepared from ^35^S-methionine-labeled proteins separated by SDS-PAGE (Figure 8B). Taken together, these results indicate that hypusine-modified eIF5A contributes to arsenite-induced re-programming of protein synthesis.

**Figure 9:**
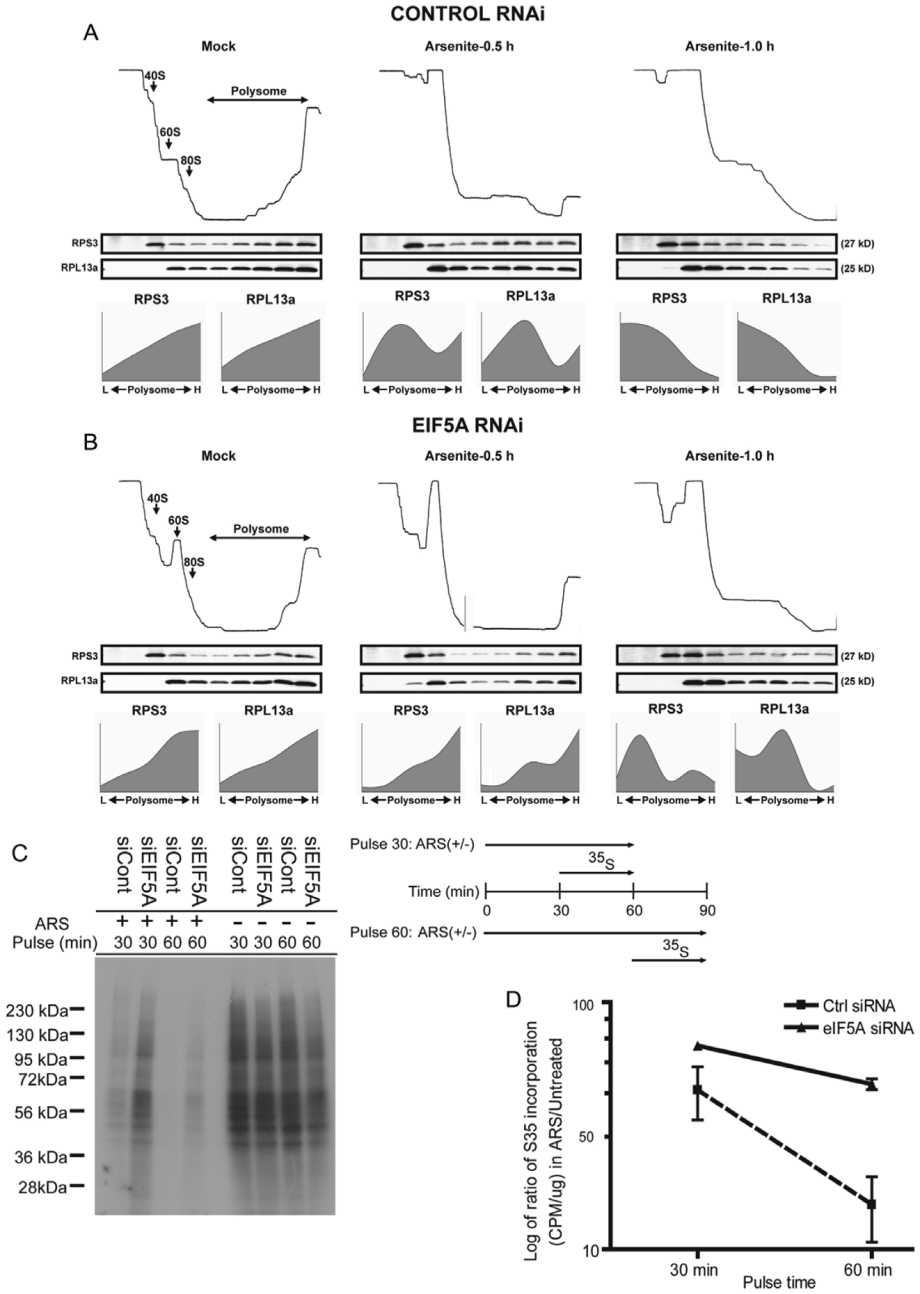
Depletion of eIF5A delays stress-induced polysome disassembly. (A) Polysome profiling analysis in U2OS cells treated with a control siRNA. Upper panels show polysome profiles obtained from a UV monitor (254 nm). Peaks corresponding to 40S, 60S and 80S ribosomes as well as polysomes are indicated with arrows. Middle panels show western blots of individual fractions across the gradient probed with antibodies against RPS3 and RPL13a. Bottom panels are graphical representations of quantitative distribution analyses of RPS3 and RPL13a in polysome fractions 6–10. The abundance of ribosomal proteins in each fraction was divided by the total abundance across the polysome fractions. (B) Polysome profiling analysis in U2OS cells treated with eIF5A-specific siRNAs. Panel descriptions are as in (A). (C) Pulse labeling with ^35^S-methionine. U2OS cells were treated with the indicated siRNAs, cultured in the absence or presence of arsenite, and pulsed with ^35^S-methionine prior to SDS-PAGE and autoradiographic analysis. In cells labeled “pulse 30”, ^35^S-methionine was added 30 minutes after the addition of arsenite. In cell labeled “pulse 60”, ^35^S-methionine was added 60 minutes after the addition of arsenite. (D) Logarithm of the ratio of ^35^S incorporation (CPM/µg) in arsenite/untreated U2OS cells after the pulse 30 or pulse 60 (n = 3, Error bars denote ± S.D).

In yeast, temperature sensitive eIF5A mutants confer a defect in translation elongation [10]. If eIF5A promotes translation elongation in stressed mammalian cells, the preservation of polysomes in cells lacking eIF5A (Figure 6A) could result from delayed ribosome run-off. To test this hypothesis, we compared polysome profiles and migration of ribosomal proteins in U2OS cells treated with control (Figure 9A) or eIF5A-specific (Figure 9B) siRNAs. In control cells, polysomes and ribosomal proteins migrate near the bottom of the gradient (Figure 9A, left panel; densitometric quantification of ribosomal proteins is plotted in the graphs labeled RPS3 and RPL13a; these profiles are restricted to the polysome-containing fractions in these gradients). With the addition of arsenite, both polysomes and ribosomal proteins are progressively shifted towards the top of the gradient (Figure 9A, middle panel: 0.5 h; right panel: 1 h), a consequence of stress-induced translational repression and ribosomal run-off. In eIF5A knockdown cells (Figure 9B), the shift of polysomes and ribosomal proteins towards the top of the gradient is significantly delayed, consistent with impaired translation elongation. These results suggest that impaired polysome disassembly and stress granule assembly in response to eIF5A knockdown results from impaired translation elongation. The enhanced protein synthesis observed in arsenite-treated cells following eIF5A knockdown (Figure 8A and 8B) occurs when ^35^S-methionine is added after 30 minutes of arsenite treatment. At this juncture, control cells have partially disassembled their polysomes (Figure 9A, middle panel), whereas eIF5A knockdown cells have nearly intact polysomes (Figure 9B, middle panel). Because of this, increased protein synthesis may be paradoxically due to a reduced ribosome transit time. To test this hypothesis, siRNA-treated U2OS cells were cultured with arsenite (0.1 mM) for 30 or 60 minutes prior to pulsing with ^35^S-methionine for 30 minutes. Cells were washed and lysates were processed for SDS-PAGE/autoradiography. Whereas knockdown of eIF5A enhances protein synthesis during the 30-minute pulse, it has a much smaller effect during the 60-minute pulse (Figure 9C and 9D). In contrast to arsenite-induced polysome disassembly, which is initiated by inhibition of translation initiation, puromycin-induced polysome disassembly is a consequence of ribosome drop-off. This mechanism should be unaffected by treatments that alter the ribosome transit time. Taken together, these results indicate that eIF5A knockdown reduces ribosome transit time to modulate stress-induced translational re-programming.

## Discussion

We have discovered that ODC, an enzyme required for the synthesis of polyamines (e.g., spermine and spermidine) [21], and eIF5A, a translation regulatory factor that is modified by polyamines [22], are required for the arsenite-induced assembly of SGs. These results are linked by the finding that deoxyhypusine synthase, an enzyme that covalently joins spermidine to eIF5A [23], also inhibits SG assembly. We conclude that hypusine-modified eIF5A is essential for the arsenite-induced assembly of SGs. These findings add polyamines to a growing list of post-translational modifications that are required for RNA granule assembly [16].

Targeted depletion of ODC, but not eIF5A, also inhibits the arsenite-induced assembly of PBs. This suggests that polyamines can regulate RNA granule assembly in an eIF5A-independent manner. As polyamines regulate diverse cellular functions, the requirement for ODC may be an indirect consequence of altered cellular metabolism. An analogous situation was observed in studies of the hexosamine signaling pathway, another regulator of RNA granule assembly. Whereas depletion of sortilin, an upstream modulator of hexosamine biosynthesis, inhibits SG and PB assembly, depletion of *O*-GlcNAc transferase, a downstream modulator of hexosamine biosynthesis, inhibits oxidative stress-induced SG, but not PB, assembly [18]. Because the polyamine synthetic pathway [24], [25] and the hexosamine biosynthetic pathway [26] are activated during stress, modifications resulting from these pathways may be more important for SG assembly than PB assembly. On the other hand, the assembly of yeast P-bodies in response to glucose deprivation requires eIF5A [10]. It remains to be determined whether the ability of eIF5A to regulate P-body assembly is determined by the species or the type of stress.

The role of eIF5A in protein synthesis has been controversial. Hypusine-modified eIF5A interacts with ribosomes and other components of the translation machinery [1], [2], [27]. In reticulocyte lysates, eIF5A promotes the synthesis of methionyl puromycin, but not globin protein, from a globin mRNA substrate [1]. In yeast expressing an unstable eIF5A chimera, global protein synthesis is reduced by 30% indicating that eIF5A is required for optimal protein synthesis [12]. This modest reduction in protein synthesis has been proposed to be due to the ability of eIF5A to support translation elongation as eIF5A binds to elongation factor eEF2 [2]. In support of this hypothesis, temperature-sensitive eIF5A mutants are hypersensitive to the peptidyltransferase inhibitors sparsomycin and anisomycin [2] and significantly reduce ribosome transit time [10], [11]. In our experiments using mammalian U2OS cells, efficient knockdown of eIF5A (>80%) reduces the rate of protein synthesis by about 5% compared to knockdown controls. A more striking effect is observed in cells subjected to arsenite-induced oxidative stress, in which the rate of ribosome run-off is significantly reduced. Thus eIF5A may be particularly important in promoting translation elongation in stressed compared to unstressed cells. As stress-induced phosphorylation of eEF2 inhibits translation elongation [28]–[30], it is possible that eIF5A interacts with phospho-eEF2 to maintain ribosome transit in cells subjected to adverse conditions. This is consistent with results in yeast showing that eIF5A and eEF2 functionally interact to promote translation elongation [11]. In U2OS cells treated with control vs. eIF5A specific siRNAs, arsenite treatment rapidly disassembles polysomes in control cells, but not in eIF5A knockdown cells. During this window, eIF5A knockdown cells have more translating ribosomes than control cells. Thus, they synthesize more protein than control cells despite the fact that the rate of ribosomal elongation is reduced. At later times, when polysome disassembly has occurred in both control and eIF5A knockdown cells, total protein synthesis is markedly reduced. These results support a role for eIF5A in translation elongation and suggest that it may play a particularly important role in stressed cells.

In unstressed cells, knockdown of eIF5A promotes the accumulation of 60S ribosomal subunits, a phenotype that is also observed in cells treated with the hypusination inhibitor GC7 and in yeast cells that are depleted of eIF5A [12], [31]. A similar phenotype is observed in cells heterozygous for the ribosome assembly factor eIF6 [20]. These results suggest that eIF5A may regulate some aspect of ribosome assembly under normal culture conditions.

Our results indicate the eIF5A has a very modest effect on protein synthesis under normal culture conditions. In contrast, knockdown of eIF5A has a profound phenotype in cells subjected to arsenite-induced oxidative stress. In these cells, polysome disassembly and stress granule assembly are significantly inhibited. Polysome profile analysis reveals that this is accompanied by delayed ribosome run-off resulting from reduced rates of ribosome transit under stress conditions. While this delays SG assembly, it probably doesn't prevent SG assembly. Thus eIF5A is probably not a SG assembly factor, *per se*. We propose that eIF5A is a non-essential translation elongation factor in mammalian cells (but possibly more “essential” in yeast [10], [11]) whose influence becomes apparent when one or more canonical elongation factors are inactivated during stress. In this capacity, eIF5A may play an important role in reprogramming protein synthesis in stressed cells, a process that can determine whether cells survive under adverse conditions.

The finding that eIF5A knockdown preserves polysomes in stressed cells is consistent with its proposed role in translation elongation. By promoting translation elongation, eIF5A may be required for the efficient disassembly of polysomes in response to stress. In cells lacking eIF5A, persistent global protein synthesis may delay the reprogramming of translation that helps cells recover from adverse environmental conditions. This function may be responsible for eIF5A-mediated effects on cell survival [7], [8], [32]. Thus, our results support previous suggestions that eIF5A promotes translation at the level of translation elongation and reveal an important consequence of this phenotype in stressed cells.

## Materials and Methods

### Cell culture, drug treatment and transfection

U2OS (human osteosarcoma), COS7, and RDG3 cells described previously [18] were maintained in DMEM (Gibco, Grand Island, NY) supplemented with 10% inactivated fetal bovine serum (Sigma), 1% (v/v) penicillin and streptomycin (Sigma) at 37°C in 6% CO_2_. To induce SGs and PBs, cells were treated with sodium arsenite (0.1∼0.5 mM) for 30 min to 1 h. Clotrimazole was used at 20 µM in serum-free media for 1 h. Cells were subjected to heat shock at 44°C for 1 h. To block hypusination of eIF5A, N1-guanyl-1,7-diaminoheptane (GC-7) [19] was used at a concentration of 10 µM for 24∼48 hrs. siRNA and Plasmids were transiently transfected using Lipofectamin 2000 (Invitrogen) and FuGene6 (Roche), respectively. *EIF5A* constructs were kindly provided by Dr. Myunghee Park from NIH.

### Antibodies

Mouse anti-eIF5A (BD Biosciences), goat anti-eIF3b (Santa Cruz Biotechnology), rabbit anti-Rck (Bethyl), rabbit anti-phospho-eIF2α (Assay Designs), rabbit anti-RPS3 (Cell signaling), rabbit anti-RPL13a (Cell signaling), mouse anti-p70 S6 kinase (Santa Cruz Biotechnology, sc-8418) for Hedls detection [33], mouse anti-αActin (Chemicon) and rabbit anti-DHS (Santa Cruz Biotechnology, sc-67161) were obtained and used for immunoblotting, immunopurification, and immunofluorescence. Cy2, Cy3, Cy5, and HRPO- conjugated secondary antibodies were purchased from Jackson Immunoresearch Labs.

### RNA interference and quantification of knockdown effect on SGs and PBs

Cells were transfected with siRNAs at 40 nM or 100 nM final concentration. After 40 h, cells were trypsinized, re-seeded and transfected again for another 40–44 h. For single transfections, cells were treated for 48 to 72 h and processed for the next step. Knockdown efficiencies were verified by either immunoblotting or quantitative RT-PCR (RT-qPCR). siRNA target sequences used in the studies are as follows: ODC1-1, GCAUUUGUAGCUUGUACAA; ODC1-2, GAGCAGACCUUUAUGUAUU; 5A-1, GCAAGGAGAUUGAGCAGAA; 5A-2, CAAUCAAGGCCAUGGCAAA; DHS-1, CGGGAUCAAUAGGAUCGGA; DHS-2, CGUAUUGGUGACCACAGCU. Control siRNAs (siCONT), D0 (GCATTCACTTGGATAGTAA) and U0 (GAATGCTCATGTTGAAUCA), were described previously [34]. Knockdown effects on SGs and PBs were assessed by quantifying the number of cells with SGs or PBs out of more than 200 cells from at least 3 different fields. For RT-qPCR total RNA samples were prepared with TRIzol (Invitrogen) and quantified by spectrophotometry. 5 µg RNA was treated with DNAase followed by cDNA synthesis using i-script cDNA synthesis kit (Bio-Rad). RT-qPCR was performed based on SYBR green method using SYBR mix (Bio-Rad) in a cycler (MX3000p; Stratagene). Beta-2-microglobulin and actin were used as internal controls, and showed similar results. Primers for RT-qPCR were purchased from SuperArray for DHS quantitation or designed by using Primer 3 software. Primer sequences are as follows: ODC1-1-F, TTCCAAAGCAGTCTGTCGTCT; ODC1-1-R, GGAAGCTGACACCAACAACAT; ODC1-2-F, GGTGATTGGATGCTCTTTGAA; ODC1-2-R, CAGGCCCTGACATCACATAGT; ACTIN-F, ACCAACTGGGACGACATGGAGAAA; ACTIN-R, TAGCACAGCCTGGATAGCAACGTA.

### Immunofluorescence microscopy

General immunofluorescence procedures were performed as previously described [33]. Cells grown on cover slips were rinsed with PBS (phosphate-buffered saline, pH 7.4), fixed with 4% paraformaldehyde for 15 min, permeabilized with cold methanol for 10 min or with 0.5% Triton-X 100 in PBS and blocked by incubation in 5% normal horse serum in PBS containing 0.1% sodium azide (NHS/PBS-A) for at least 1 h. Primary antibodies diluted in blocking solution were added and incubated for ∼1 h at room temperature. Cells were then washed twice with PBS. Finally, cells were incubated with secondary antibodies (Jackson Immunoresearch ML grade) for 1 h, washed with PBS (3x, 5–10 min each), and mounted in polyvinyl-based mounting media. Widefield fluorescence microscopy was performed using an Eclipse E800 (Nikon) equipped with epifluorescence optics and a digital camera (CCD-SPOT RT, Diagnostic Instruments). The images were compiled using Adobe Photoshop (v.CS). To detect endogenous eIF5A (BD Biosciences), cells were fixed with 4% paraformaldehyde and permeabilized with 0.5% Triton-X100 in PBS for 10 min.

### Western blot analysis

Cell extracts were prepared with RIPA buffer (25 mM Tris-HCl, pH 7.6, 150 mM NaCl, 1% NP-40, 1% sodium deoxycholate, 0.1% SDS) or direct lysis buffer (5 mM MES at pH 6.5, 2% SDS) and proteins were quantified, where indicated, with BCA assay reagent (Pierce). Total proteins (5–10 µg) were subjected to 4–20% gradient PAGE in SDS, transferred to nitrocellulose membranes and probed with the indicated antibodies using PBS containing 5% normal horse serum as a blocking reagent.

### Polysome profiling analysis

All procedures with siRNA treated cells were followed as previously described [18]. U2OS cells (1∼2×10^6^) were plated and treated with GC-7 (10 µM) or vehicle control for 24∼48 hrs. Before harvest, cells were incubated with or without 0.1 mM sodium arsenite for 1 h prior to addition of 10 µg/ml cyclohexamide, washed with cold PBS, then scrape harvested into 1 ml polysome lysis buffer (15 mM Tris-HCl, pH 7.4, 15 mM MgCl_2_, 0.3 M NaCl, 1% Triton X-100) supplemented with 0.1% (v/v) beta-mercaptoethanol, 100 µg/ml cyclohexamide, 0.1 mg/ml heparin, protease inhibitor cocktail (EDTA free, PIERCE), and RNAsin (Ambion). After 15 min tumbling in the cold room, nuclei were pelleted (10,000 g, 10 min) and supernatants were fractionated over 10–50% linear sucrose gradients using ultracentrifugation (35,000 rpm, 3 h 10 min) in a Beckman ultracentrifuge and an SW40 Ti rotor. Gradients were eluted using a gradient fractionator (Brandel) and monitored with a UA-5 detector (ISCO). Fractions were acetone-precipitated, and processed for further analysis.

### ^35^S metabolic labeling

U2OS cells were washed once with PBS and once with labeling medium (cysteine and methionine-free DMEM with 10% dialyzed FBS). Then the cells were starved with labeling medium with or without sodium arsenite (100 µM) for 0.5 hour or 1 hour. After that cells were incubated with fresh labeling medium containing 100 µCi per mL of ^35^S at 37°C with or without sodium arsenite (100 µM) for 0.5 h. After labeling, cells were washed with cold PBS once and then harvested in the lysis buffer (20 mM HEPES, pH 7.4, 2% SDS). The ^35^S incorporation rate (CPM/µg protein) was measured after acetone precipitation.

### IEF gel analysis

Cells were harvested with RIPA buffer following standard methods. After sonication, 30 µg of total protein was precipitated with Trichloroacetic acid (TCA) and then resuspended in 15 µl of Invitrogen IEF sample buffer (pH 3–7). Resuspended proteins were analyzed on an IEF gel (pH 3–7) from Invitrogen following the manufacturer's instructions. After electrophoresis, the gel was transferred to nitrocellulose membrane and developed using a mouse anti-human eIF5A antibody.

## Supporting Information

Figure S1 Figure S1A. Effects on PB formation in cells transfected with control or eIF5A-specific siRNAs (Figure 2B). Cell counting data are combined from different experiments in mock and arsenite treated conditions and expressed as a mean ± S.D. Figure S1B. U2OS cells were cultured in the absence or presence of GC7 (20 µM) for 24 or 48 hours then cultured in the absence (untreated) or presence (ARS) of sodium arsenite (100 µM, 1 hour). Cells were processed for immunofluorescence microscopy using antibodies reactive with RCK/p54, Hedls, or eIF3b. Merged views are shown at the right. Scale bar, 10 µm.
(5.82 MB TIF)

Figure S2 U2OS cells were transfected with control (siCONT) or eIF5A-targeted siRNAs (siEIF5A-1 and siEIF5A-2) prior to culturing in media alone (Mock), or media containing sodium arsenite (arsenite; 100 µM, 1 hour), thapsagargin (TG; 1 µM, 1 hour), pateamine A (PA; 50 nM, 1 hour) or clotrimazole (CZ; 20 µM, 1 hour). Cells were then processed for immunofluorescence microscopy using anti-eIF3b to visualize SGs. Scale bar, 10 µm.
(5.82 MB TIF)
