# Supplementary figures and images for: eIF5A Promotes Translation Elongation, Polysome Disassembly and Stress Granule Assembly
Source: PLoS One. 2010 Apr 1;5(4):e9942. doi: 10.1371/journal.pone.0009942 (PMC2848580; doi:10.1371/journal.pone.0009942)

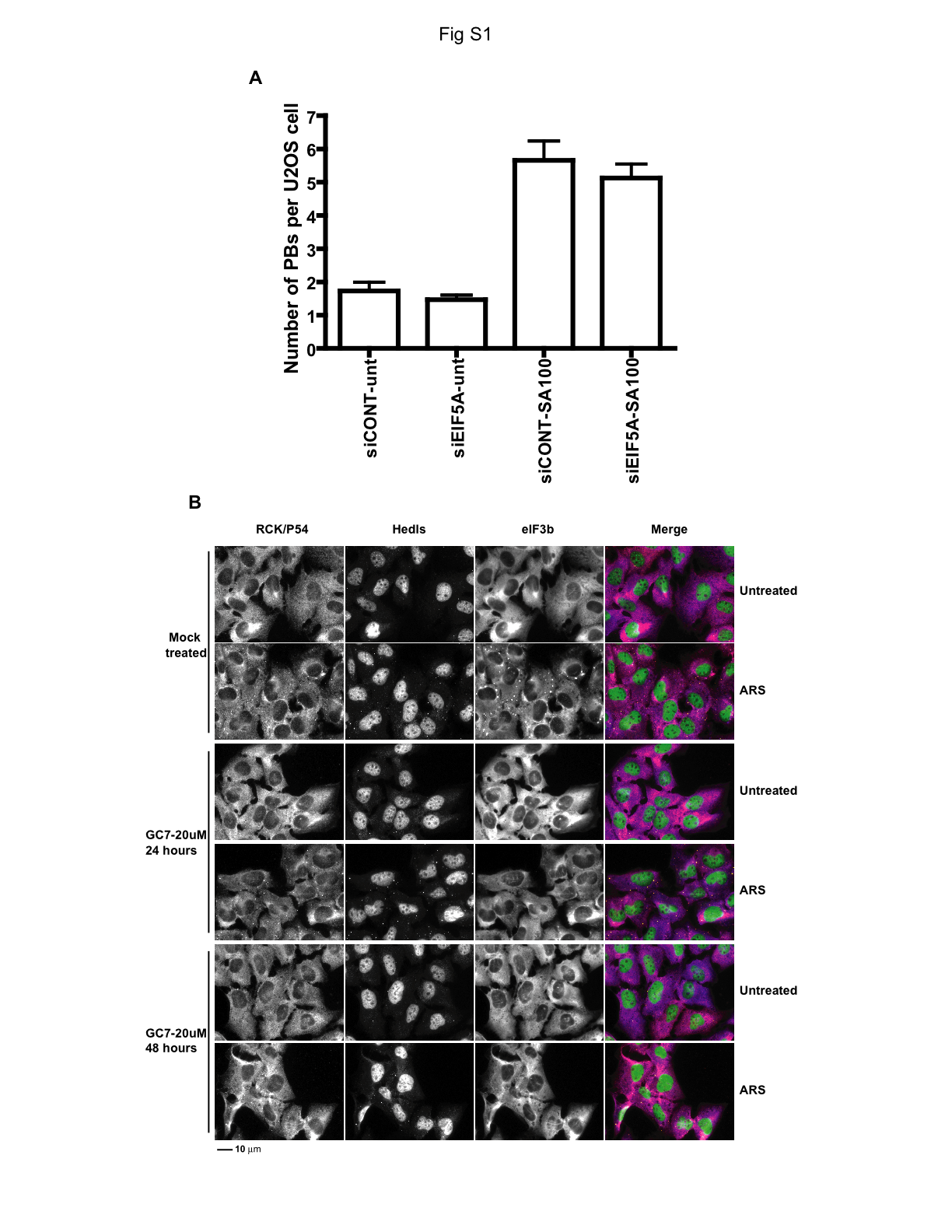

Supplement: Figure S1 — A. Effects on PB formation in cells transfected with control or eIF5A-specific siRNAs (Figure 2B). Cell counting data are combined from different experiments in mock and arsenite treated conditions and expressed as a mean ± S.D. Figure S1B. U2OS cells were cultured in the absence or presence of GC7 (20 µM) for 24 or 48 hours then cultured in the absence (untreated) or presence (ARS) of sodium arsenite (100 µM, 1 hour). Cells were processed for immunofluorescence microscopy using antibodies reactive with RCK/p54, Hedls, or eIF3b. Merged views are shown at the right. Scale bar, 10 µm. (5.82 MB TIF) [file pone.0009942.s001.tif]

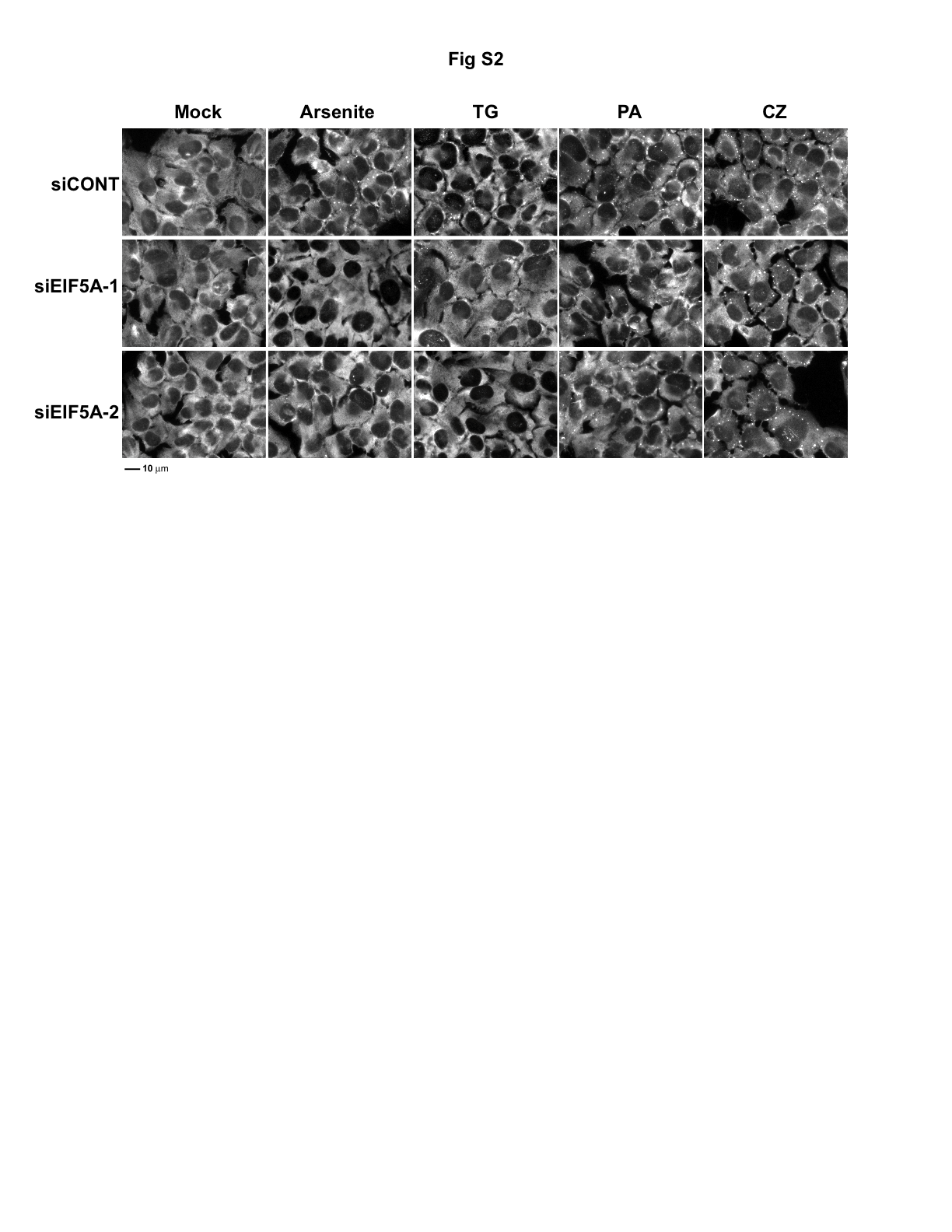

Supplement: Figure S2 — U2OS cells were transfected with control (siCONT) or eIF5A-targeted siRNAs (siEIF5A-1 and siEIF5A-2) prior to culturing in media alone (Mock), or media containing sodium arsenite (arsenite; 100 µM, 1 hour), thapsagargin (TG; 1 µM, 1 hour), pateamine A (PA; 50 nM, 1 hour) or clotrimazole (CZ; 20 µM, 1 hour). Cells were then processed for immunofluorescence microscopy using anti-eIF3b to visualize SGs. Scale bar, 10 µm. (5.82 MB TIF) [file pone.0009942.s002.tif]
